# The Use of Q-ICPMS to Apply Enriched Zinc Stable Isotope Source Tracing for Organic Fertilizers

**DOI:** 10.3389/fpls.2019.01382

**Published:** 2019-11-13

**Authors:** Thilo Dürr-Auster, Matthias Wiggenhauser, Christophe Zeder, Rainer Schulin, Dominik J. Weiss, Emmanuel Frossard

**Affiliations:** ^1^Group of Plant Nutrition, Department of Environmental System Science, Institute for Agricultural Sciences, ETH Zurich, Zurich, Switzerland; ^2^Geochemistry Group, Institut des Sciences de la Terre, CRNS, Université Grenoble-Alpes, Grenoble, France; ^3^Laboratory of Human Nutrition, Department of Health Sciences and Technology, Institute of Food, Nutrition and Health, ETH Zurich, Zurich, Switzerland; ^4^Soil Protection, Department of Environmental System Science, Institute of Terrestrial Ecosystems, ETH Zurich, Zurich, Switzerland; ^5^Environmental Geochemistry, Department of Earth Science and Engineering, Imperial College London, London, United Kingdom

**Keywords:** zinc, stable isotopes, isotope dilution, labelling, soil, organic fertilizer, source tracing, ryegrass

## Abstract

Organic fertilizer applications can contribute to Zinc (Zn) biofortification of crops. An enriched stable isotope source tracing approach is a central tool to further determine the potential of this biofortification measure. Here, we assessed the use of the widely available quadrupole single-collector ICPMS (Q-ICPMS, analytical error = 1% relative standard deviation) and the less accessible but more precise multicollector ICPMS as reference instrument (MC-ICPMS, analytical error = 0.01% relative standard deviation) to measure enriched Zn stable isotope ratios in soil–fertilizer–plant systems. The isotope label was either applied to the fertilizer (direct method) or to the soil available Zn pool that was determined by isotope ratios measurements of the shoots that grew on labeled soils without fertilizer addition (indirect method). The latter approach is used to trace Zn that was added to soils with complex insoluble organic fertilizers that are difficult to label homogeneously. To reduce isobaric interferences during Zn isotope measurements, ion exchange chromatography was used to separate the Zn from the sample matrix. The ^67^Zn:^66^Zn isotope ratios altered from 0.148 at natural abundance to 1.561 in the fertilizer of the direct method and 0.218 to 0.305 in soil available Zn of the indirect method. Analysis of the difference (Bland–Altman) between the two analytical instruments revealed that the variation between ^67^Zn:^66^Zn isotope ratios measured with Q-ICPMS and MC-ICPMS were on average 0.08% [95% confidence interval (CI) = 0.68%]. The fractions of Zn derived from the fertilizer in the plant were on average 0.16% higher (CI = 0.49%) when analyzed with Q- compared to MC-ICPMS. The sample matrix had a larger impact on isotope measurements than the choice of analytical instrument, as non-purified samples resulted on average 5.79% (CI = 9.47%) higher isotope ratios than purified samples. Furthermore, the gain in analytical precision using MC-ICPMS instead of Q-ICPMS was small compared to the experimental precision. Thus, Zn isotope measurements of purified samples measured with Q-ICPMS is a valid method to trace Zn sources in soil–fertilizer–plant systems. For the indirect source tracing approach, we outlined strategies to sufficiently enrich the soil with Zn isotopes without significantly altering the soil available Zn pool.

## Introduction

Low Zn concentrations in edible plant parts can lead to Zn deficiency in humans especially in areas with cereal based diets (Black et al., 2008, Wessells and Brown, 2012). Thus, there is a need for agricultural measures that increase Zn concentrations in staple foods such as the addition of inorganic and organic fertilizers to soils (Aghili et al., 2014; Cakmak and Kutman, 2018).

Isotope source tracing allows to precisely determine the efficiency of fertilizers by determining the contributions of fertilizers and soil to plant Zn uptake (McBeath et al., 2013). There are two ways of doing this. In the direct method, the fertilizer itself is labeled with an isotope tracer before being added to the unlabeled soil. In the indirect method, soluble Zn (e.g. ZnCl_2_) is added to the soil to label the plant available Zn fraction of the soil and the subsequent addition of an unlabeled fertilizer to the soil leads then to an isotope dilution of the plant available Zn fraction. For both source tracing techniques, three conditions need to be fulfilled: i) the introduced spike should not alter the availability of Zn from the labeled source, ii) the isotope used for labeling do not differ in their behavior in the system, iii) and the isotopic label should be homogeneously mixed in the source to be traced (Cobelli et al., 2000; Hamon et al., 2008). The indirect method is particularly useful for complex insoluble fertilizers (organic and mineral) that are difficult to label homogeneously (Frossard et al., 2011). It was successfully used to determine the effects of complex insoluble fertilizers on N and P nutrition of crops (Douxchamps et al., 2011; Nanzer et al., 2014) and to measure Zn uptake from Zn oxide (McBeath and McLaughlin, 2014).

Zinc source tracing can employ natural stable isotope fractionation, radioisotopes or enriched stable isotopes. The radioisotope ^65^Zn has successfully been used in soil–plant systems with both source tracing approaches (Sinaj et al., 1999; Diesing et al., 2008; Aghili et al., 2014; McBeath and McLaughlin, 2014; Hippler et al., 2015). Compared to stable isotope systems, radioisotope systems are more readily analyzed (Stürup et al., 2008; Wiggenhauser et al., 2018). However, ^65^Zn is a gamma-emitter with a half-life of 244.4 days and safety issues, legal regulation limits for *in-situ* experiments, transport as well as waste management and storage are problematic and complicate experiments. In the last decades, technical improvements of mass spectrometry enabled to measure natural variations of metal stable isotopes ratios (Weiss et al., 2008; Wiederhold, 2015). To this end, a multicollector ICPMS (MC-ICPMS) is used that achieves a precision of 0.01% relative standard deviation (RSD) for isotope ratio measurements which is adequate to study natural biogeochemical fractionation processes (Cloquet et al., 2008; Caldelas and Weiss, 2017). Analysis of such natural variations has allowed source tracing of metals on field or catchment scales (Borrok et al., 2009; Bigalke et al., 2010; Imseng et al., 2019; Yang et al., 2019). However, this approach requires that the isotope ratios of sources and sinks are distinguishable, which is not always the case (Wiggenhauser et al., 2018).

The addition of enriched stable isotopes to fertilizers or soils makes it easier to distinguish the isotope ratios of these sources and consequently, the demand on analytical precision is reduced, which allow to use a single-collector quadrupole ICP-MS equipped with a collision cell (Q-ICPMS) with a typical precision ∼ 1% RSD (Stürup et al., 2008). Compared to MC-ICPMS, these instruments are less expensive and more accessible since they are routinely used to measure elemental concentrations. Furthermore, the less complex and demanding measurement procedure permits increased sample throughput. Compared to radioisotopes, the use of enriched metal isotopes does not require special waste management and a laboratory that fulfills the safety requirements. However, the use of stable isotopes requires the addition of much larger masses of isotopic label to the labeled source than the use of radioisotopes (Stürup et al., 2008).

The precision of mass spectrometry isotope measurements can be compromised by isobaric interferences (Mason et al., 2004a) and mass discrimination effects (Mason et al., 2004b). Isobaric interferences are due to elemental isobars (species having the same mass as the measured isotope), originating from instrumental and sample matrix components (Mason et al., 2004a). These interferences can be minimized by separating the target element from the sample matrix by means of resin ion exchange chromatography (i.e. sample purification, Durrant et al., 1994; Mason et al., 2004a). Mass discrimination effects are due to mass-dependent fractionation within the instrument and can be corrected by using a sample-standard bracketing method (Walczyk, 2001; Mason et al., 2004b).

McBeath and co-workers (2013) used Q-ICPMS to assess the Zn efficiency of a Zn coated P fertilizer enriched with the stable ^67^Zn isotope. To our knowledge, there is no study yet showing that Q-ICPMS can be used in combination with the indirect method and stable Zn isotope labeling. This is particularly interesting though, since several studies showed that organic fertilizer can increase the Zn concentration in crops (Amer et al., 1980; Manzeke et al., 2012; Aghili et al., 2014; Habiby et al., 2014; Soltani et al., 2014; Grüter et al., 2017; Manzeke et al., 2017). An efficient indirect Zn source tracing technique would allow quantification of the Zn contribution of organic fertilizers to the plant. However, compared to the direct source tracing approach, the isotope enrichment in the labeled source is less strong since the isotope label that is added to soil is mixed with the Zn in the soil which has Zn isotopes that are at natural abundance. Hence, for the indirect method, the precision of the Q-ICPMS could become an issue.

The objective of this study was to assess whether the precision of Q-ICPMS for measuring stable Zn isotope ratios is adequate to determine the transfer of Zn from complex organic fertilizer to plant in systems spiked with ^67^Zn without compromising the conditions outlined before. To this end, Italian ryegrass (*Lolium multiflorum*) was grown under controlled conditions and fertilized with a straw compost. We investigated the Zn transfer from the compost into the plants in two types of treatments: i) With a ^67^Zn labeled compost in a non-labeled soil (direct method) and ii) with a non-labeled compost added to a soil labeled with ^67^Zn (indirect method). The Q-ICPMS measurements were compared with MC-ICPMS measurements whereby the MC-ICPMS was considered as a reference method.

## Material and Methods

### Soils

Two arable soils from Switzerland one from Heitenried and one from Lindau, were chosen for this study (**Table 1**). The soils differed in pH (Heitenried 4.9, Lindau 7.7) and total Zn content (Heitenried 54.1 mg kg^−1^, Lindau 101 mg kg^−1^). On both sites, the soils were collected from a depth of 0–20 cm and sieved to 7 mm aggregate size. The soil batches used for the indirect method were labeled with ^67^Zn using the following procedure: four boxes were filled with a layer of 20 cm of soil above a 30 cm layer of foam glass drainage gravel. The two layers were separated from each other by a fleece mat. Two boxes were filled with Heitenried and two with Lindau soil. One box with each soil was then percolated for 20 weeks with ^67^Zn-enriched nutrient solution. For the direct method, the other two boxes were treated in the same way with non-enriched solution. The ^67^Zn-enriched nutrient solutions were obtained from a project that determined bioavailability of Zn in wheat meals (Signorell et al. 2019). For that study, wheat was grown hydroponically in ^67^Zn-enriched and non-enriched nutrient solutions that were frequently replaced. The left overs of the hydroponic nutrient solutions were added to the soils of the experiment here by using 18 randomly distributed nozzles. The nutrient solutions percolated through the soils by gravity and were recollected underneath the soil and again added to the soil through the nozzles. The nozzles were frequently randomized after the soils were carefully mixed by hand. The average ^67^Zn isotope enrichment of the nutrient solution was equal to the isotope enrichment in the hydroponically grown wheat (^67^Zn abundance = 32%, Signorell et al. 2019). After the labeling procedure, the four soil batches were air-dried, homogenized using a concrete mixer for minimum 10 min, sieved to 2 mm, and stored for 1 year, until they were used for the study presented here. The Zn isotope composition of the total soil Zn was determined after the labeling.

**Table 1:** Soil properties (labeled soil).

Origin	Heitenried Switzerland	Strickhof Switzerland
FAO classification^a^	Fluvisol	Cambisol
Clay (g kg^−1^)^b^	146	202
Silt (g kg^−1^)^b^	235	344
Sand (g kg^−1^)^b^	619	454
pH_H2O_^c^	4.9	7.7
Zn DTPA (mg Zn kg^−1^)^d^	4.1	5.2
Zn total (mg Zn kg^−1^)^e^	54.1	101
^67^Zn-total (%, mol/mol)^f^	4.98	4.47
WHC_max_ (g H_2_O kg^−1^)^h^	387	447

a IUSS Working Group WRB. 2015. World Reference Base for Soil Resources 2014, update 2015. International soil classification system for naming soils and creating legends for soil maps. World Soil Resources Reports No. 106. Food and Agricultural Organization of the United Nations (FAO), Rome.

b Gravimetric measurement.

c pH in H_2_O with 1:2.5 solid:liquid ratio.

d Diethylenetriamine pentaacetic acid (DTPA) extractable Zn (Lindsay and Norvell, 1978).

e Energy dispersive X-ray fluorescence spectrometry.

f HNO_3_ microwave digested Zn fraction of the soil.

h Soil saturation with H_2_O without external pressure. Water Holding Capacity (WHC)

### Wheat Straw Composts

The ^67^Zn-labeled and non-labeled wheat straws used to produce the organic fertilizer were obtained from the same hydroponic experiment from which the nutrient solutions were obtained (Signorell et al. 2019). Four kilograms of each straw type were hashed and composted at 40°C during 4 months. To accelerate microbial decomposition, we added ammonium nitrate and water (>18.2 MΩ) at regular intervals and mixed the composting straw after each addition. After incubation, the composts were dried at 65°C and finely ground.

### Growth Trial

Ryegrass was grown in pots containing 400 g dry soil. For direct source tracing, non-labeled soil was mixed with ^67^Zn-labeled wheat straw compost, whereas for indirect source tracing ^67^Zn-labeled soil was mixed with non-labeled wheat straw compost. The compost was added as low and high dose (**Table 2**). Reference treatments without Zn compost were established with labeled and unlabeled soils for control. The control soils were used to provide the isotope composition of the plant available Zn pool of the soil. Furthermore, we included a treatment with ^66^Zn soil labeling to widen the range of ^67^Zn:^66^Zn available for analysis. All treatments were prepared with four treatment replicates. The plants were grown in a climate chamber with a daily photoperiod of 14 h at 25 klx. Temperature and relative humidity were set to 24°C and 60% during daytime and to 18°C and 65% during nighttime, respectively.

**Table 2:** Experimental parameters.

Method	Treatment name	Stable isotope label	Zn fertilizer type	Zn content amendment	Application rate^a^	Zn input
				**mg kg** **^−1^**	**g kg** **^−1^** **soil**	**mg kg** **^−1^** **soil**
Direct	Reference	^67^Zn	None	−	−	−
	High direct	^67^Zn	Wheat straw compost	32.4	33	1.069
	Low direct	^67^Zn	Wheat straw compost	32.4	12.5	0.405
	ZnSO_4_	^66^Zn	ZnSO_4_	4.4*10^5^	4.49*10^−7^	0.102
Indirect	Reference	^67^Zn	None	−	−	−
	Low indirect	^67^Zn	Wheat straw compost	64	12.5	0.8
	High indirect	^67^Zn	Wheat straw compost	64	33	2.112

a “Low” and the “high” application rates correspond to an application of 32.5 and 85.8 t ha−^1^ organic fertilizer in the field, respectively (assuming a soil ploughing depth of 20 cm and a soil density of 1.3 t m−^3^). A typical application rate of organic fertilizer is 5 t ha−^1^ (Grüter et al. 2017). The comparably high application rates of this study were chosen to create a large range of plant Zn isotope ratios that were distinguishable from the isotope ratios of soil and fertilizer.

Each pot was sown with 0.5 g of Italian Ryegrass (*L. multiflorum*, var. Gemini). Nutrient solution was applied 14 days after sowing (DAS) at a rate of 460 mg N kg^−1^ (ammonium nitrate), 57 mg P kg^−1^, 144 mg K kg^−1^, 32 mg S kg^−1^, 50 mg Mg kg^−1^, 6 mg Fe kg^−1^ soil, 232 µg B kg^−1^, 127 µg Mn kg^−1^, 31 µg Cu kg^−1^, and 54 µg Mo kg^−1^ soil. In the treatments with low compost dose N addition was reduced to 321 mg N kg^−1^ soil and in the treatments with high compost dose to 92 mg N kg^−1^ soil for “high direct” and “high indirect” in order to supply the same total amount of N to each pot. The pots were watered to maintain a soil moisture between 40% and 80% water holding capacity of the soil. The shoots were harvested at 21 DAS (first cut) and 30 DAS (second cut), but only the samples of the second cut were used for analysis. For the first cut, Zn derived from the ryegrass seeds could significantly contribute to the total Zn uptake in ryegrass (Nanzer, 2012). Thus, the seeds could be a third Zn source with an isotope composition at natural abundance that decreases the variability of Zn isotope ratios in the ryegrass which was not desirable for this study.

### Sample Processing and Ion Exchange Chromatography

Composted wheat straw samples collected just before preparing the soil–compost mixtures and the ryegrass shoot samples collected at harvest were dried for 48 h at 65°C in bags made of pure cellulose (pergamin) and milled in tungsten bowls. Subsamples of 200 mg dry material for each treatment replicate were suspended in 2 ml H_2_O (> 18.2 MΩ) and 2 ml HNO_3_ (Rotipuran^®^ Supra 15.5 M, Zn < 0.5 µg L^−1^) in single-use glass tubes and digested in a high-pressure single-reaction microwave chamber (turboWave, MWS microwave system). An aliquot of the extract was used for total Zn analysis by means of inductively coupled plasma atomic emission spectrometry. The rest of each extract was transferred into PTFE containers, evaporated to dryness and dissolved in 6 M HCl (Rotipuran®Supra 11.6 M, Zn < 1 µg L^−1^). Zinc was separated from the matrix by ion exchange chromatography following a slightly modified protocol from Pinna et al. (2001). Spectra/Chrom^®^ Minicolumns PP, 7.5 ml with 45 µm filter were filled with 2.3 cm of anion exchange resin (AG^®^ 1-X8, 100–200 mesh, chloride form, Bio-Rad laboratories). The resin was cleaned by adding successively 3 × 5 ml HNO_3_ 2 M, 2 ml H_2_O, and 5 ml of HCl 0.5 M. Then the column was conditioned with 2 × 5 ml HCl 6 M and the samples were loaded onto the resin. Next, the matrix cations (except Cu, Fe, and Zn) were washed with 2 × 5 ml HCl 6M. Cu and Fe were eluted with 5 ml HCl 2.5 M and 3 × 5 ml HCl 0.5 M, respectively. In the last step, Zn was eluted into PTFE container with 2 × 5 ml HCl 0.005 M. The resulting eluate was evaporated to dryness, dissolved in HNO_3_ 15.5 M, and evaporated twice again. Finally, the dry residues were dissolved in 2 ml HNO_3_ 0.05 M and stored in polyethylene (PE) tubes. In order to quantify the additional benefit of ion exchange chromatography on the spectral interferences the ^67^Zn:^66^Zn ratio of the plant extracts was measured on the Q-ICPMS before and after purification.

### Sample Analysis

All purified extracts were analyzed for Zn isotope composition by means of both Q-ICPMS and MC-ICPMS. For Q-ICPMS we used an Agilent 7500ce with a helium supplied octopole reaction system. Each sample was analyzed 12 times. For each mass (^64^Zn, ^66^Zn, ^67^Zn, ^68^Zn, ^70^Zn) a three-point peak pattern was selected with an integration time of 0.1 s per point. Mass bias correction was performed by using standard-sample-standard bracketing with a conventional zinc sulfate heptahydrate solution (ZnSO_4_•7H_2_O, Sigma Aldrich) and applying a power law mass fractionation correction (Peel et al., 2009). The samples were diluted to obtain a concentration of approximately 200 µg L^−1^ in accordance with the ZnSO_4_-standard. The average of the RSD was <1.3% for all isotope ratios (^67^Zn:^xx^Zn) except ^67^Zn:^70^Zn which reached an average RSD of 2.7% due to the low natural abundance of ^70^Zn (0.61% mole fraction, Berglund and Wieser, 2011). For MC-ICPMS we used a double focusing high resolution Thermo-Finnigan Neptune (Bremen, Germany), equipped with an Apex desolvation unit (Elemental Scientific, Omaha, USA). Zinc isotopic ratios were analyzed in three blocks of 20 measurements for each sample. To correct for instrumental mass bias, standard-sample-standard bracketing was applied, using a commercial Zn standard solution (Titrisol, Merck Chemicals), where one block of 20 measurements of standard Zn was performed before and one after each block of sample measurements. Zn concentrations of samples and standard were matched (approximately 1 mg L^−1^). To correct for isobaric interference of ^64^Ni on ^64^Zn also ^62^Ni was measured. No other ratio normalizations were carried out. Regarding the analytical error, the RSD was <0.05% for all isotopic ratios. The correction factor of the standard-sample-standard bracketing was calculated based on the natural Zn isotope abundances (mole fraction in %) reported by the International Union of Pure and Applied Chemistry: 49.17% for ^64^Zn, 27.73% for ^66^Zn, 4.04% for ^67^Zn, 18.45% for ^68^Zn, and 0.61% for ^70^Zn (Berglund and Wieser, 2011). Typical procedural blanks were 1% of the total Zn in a sample.

### Source Tracing Calculation System

In the present study, the source tracing system included two Zn sources, i.e. soil available Zn and the compost, and a sink which represents the Zn isotope mixture of both sources present in the ryegrass shoots of the Zn-fertilized treatments. The terms of the mass balance formula are composed of the ^67^Zn:^66^Zn ratio of the plant and the ^67^Zn and ^66^Zn abundances (mole fraction in %) of the two sources. Consequently, all stable Zn isotope ratios (^67^Zn:^64^Zn, ^67^Zn:^66^Zn, ^67^Zn:^68^Zn, ^67^Zn:^70^Zn) of the sources needed to be measured in order to calculate the isotope abundances. The ryegrass shoots of reference treatments provided the mean isotope composition of the available Zn in the soil. The mean isotope composition of the compost was obtained from the measurement of four independently processed compost subsamples.

To calculate the fractions of plant Zn derived from the organic fertilizer, we adapted equations from McBeath et al. (2013) according to terms in **Table 3**. The source tracing system consisted of two Zn sources, i.e. soil and compost, and one sink, i.e. the ryegrass shoots. Thus, plant Zn is composed of a fraction derived from the compost (Zndf_fertilizer_) and a fraction derived from the soil (Zndf_soil_):

**Table 3:** Terminology of the mass balance equations described in Eq. 1-6.

Terms	Definition	Units
Zn_plant_	Total Zn plant shoot uptake of the Zn fertilized treatments derived from the fertilizer and the soil (equals 1)	(mole mole^−1^)
Zndf_fert_	Fraction of Zn derived from the fertilizer	(mole mole^−1^)
Zndf_soil_	Fraction of Zn derived from the soil	(mole mole^−1^)
^66^Zn_fert_	^66^Zn abundance of the fertilizer source	(mole fraction in %)
^67^Zn_fert_	^67^Zn abundance of the fertilizer source	(mole fraction in %)
^66^Zn_soil_	^66^Zn abundance of the soil source measured in the plant shoot of the reference treatments	(mole fraction in %)
^67^Zn_soil_	^67^Zn abundance of the soil source measured in the plant shoot of the reference treatments	(mole fraction in %)
^67^Zn/^66^Zn	^67^Zn:^66^Zn isotope ratio of the plant shoot grown of the fertilized treatments	(mole mole^−1^)
Zndf_fertilizer%_	Percentage of Zn in the plant shoot derived from the fertilizer	(%)

The Zn mass (µg) in the plant shoots from the Zn fertilized treatments is composed of Zn derived from the compost (Zndf_fert_) and from soil available Zn (Zndf_soil_):

(1) $$Zn_{plant}=Zndf_{fert}+Zndf_{soil}$$

The mass balance can be deconstructed with regard to ^66^Zn and ^67^Zn:

(2) $${\;}^{66}\text{​}Zn_{plant}=Zndf_{fert}*{\;}^{66}\text{​}Zn_{fert}+Zndf_{soil}*{\;}^{66}\text{​}Zn_{soil}$$

(3) $${\;}^{67}\text{​}Zn_{plant}=Zndf_{fert}*{\;}^{67}Zn_{fert}+Zndf_{soil}*{\;}^{67}\text{​}Zn_{soil}$$

The isotope ratio in the plant is obtained by dividing Eq. 3 by Eq. 2:

(4) $${\left(\frac{{\;}^{67}\text{​}Zn}{{\;}^{66}\text{​}Zn}\right)}_{plant}=\frac{Zndf_{fert}*{\;}^{67}\text{​}Zn_{fert}+Zndf_{soil}*{\;}^{67}\text{​}Zn_{soil}}{Zndf_{fert}*{\;}^{66}\text{​}Zn_{fert}+Zndf_{soil}*{\;}^{66}\text{​}Zn_{soil}}$$

Using Eq. 1, Zndf_soil_ is substituted by (Zn_plant_ − Zndf_fert_):

(5) $${\left(\frac{{\;}^{67}\text{​}Zn}{{\;}^{66}\text{​}Zn}\right)}_{plant}=\frac{Zndf_{fert}*{\;}^{67}\text{​}Zn_{fert}+(Zn_{plant}-\;Zndf_{fert})*{\;}^{67}\text{​}Zn_{soil}}{Zndf_{fert}*{\;}^{66}\text{​}Zn_{fert}+(Zn_{plant}-\;Zndf_{fert})*{\;}^{66}\text{​}Zn_{soil}}$$

As Zn_plant_ represents the total Zn in the plant shoots it can be replaced by 1. By solving the equation for Zndf_fert_ we obtain the Zn fraction derived from the fertilizer (%):

(6) $$Zndf_{fertilizer\%}=\left(\frac{{\;}^{66}\text{​}Zn_{soil}*{\left(\frac{{\;}^{67}\text{​}Zn}{{\;}^{66}\text{​}Zn}\right)}_{plant}-{\;}^{67}\text{​}Zn_{soil}}{\left({\;}^{67}\text{​}Zn_{fert}{-}^{67}\text{​}Zn_{soil}\right)-{\left(\frac{{\;}^{67}\text{​}Zn}{{\;}^{66}\text{​}Zn}\right)}_{plant}*\left({\;}^{66}\text{​}Zn_{fert}-{\;}^{66}\text{​}Zn_{soil}\right)}\right)*\;100$$

### Statistical Comparison of the Analytical and Experimental Precision

For the results, we considered three different types of precision: i) the analytical precision, which originated from repeat measurements of a single sample (e.g. isotope ratio in a single plant), ii) precision resulting from measurements of processing replicates which originated from the same sample that was processed several times (e.g. isotope ratio of the compost), and iii) experimental precision which originated from treatment replicates that consisted of n = 4 independent replicates (e.g. the mean isotope ratio of plants that grew in pots representing a treatment of the growth trial).

To compare the performance of the two analytical instruments used, we used Bland–Altman graphs computed using the software package R (Version 3.3.2, R Foundation, Vienna, Austria) and the package “BlandAltmanLeh” (Lehnert, 2015). This approach illustrates on the x-axes mean values of e.g. isotope ratios obtained with two different analytical methods and on the y-axes the absolute difference of these values. The graphical output of this approach enables to inspect trends between the two analytical techniques (e.g. higher isotope ratios deviate more than low isotope ratios) while the data output gives the mean (bias) and the 95% confidence interval of the absolute difference of the two analytical methods (Altman and Bland, 1983; Giavarina, 2015; Jarosch et al., 2015).

Differences in mean ^67^Zn abundances in the plant available soil Zn pool and in the labeled composts between Q-ICPMS and the MC-ICPMS analyses were tested using a pairwise t-test with Bonferroni p-value adjustment. Data distribution was assessed for normality using Bland–Altman plots in combination with the Shapiro–Wilk test.

## Results

### Source Tracing System

**Figure 1:**
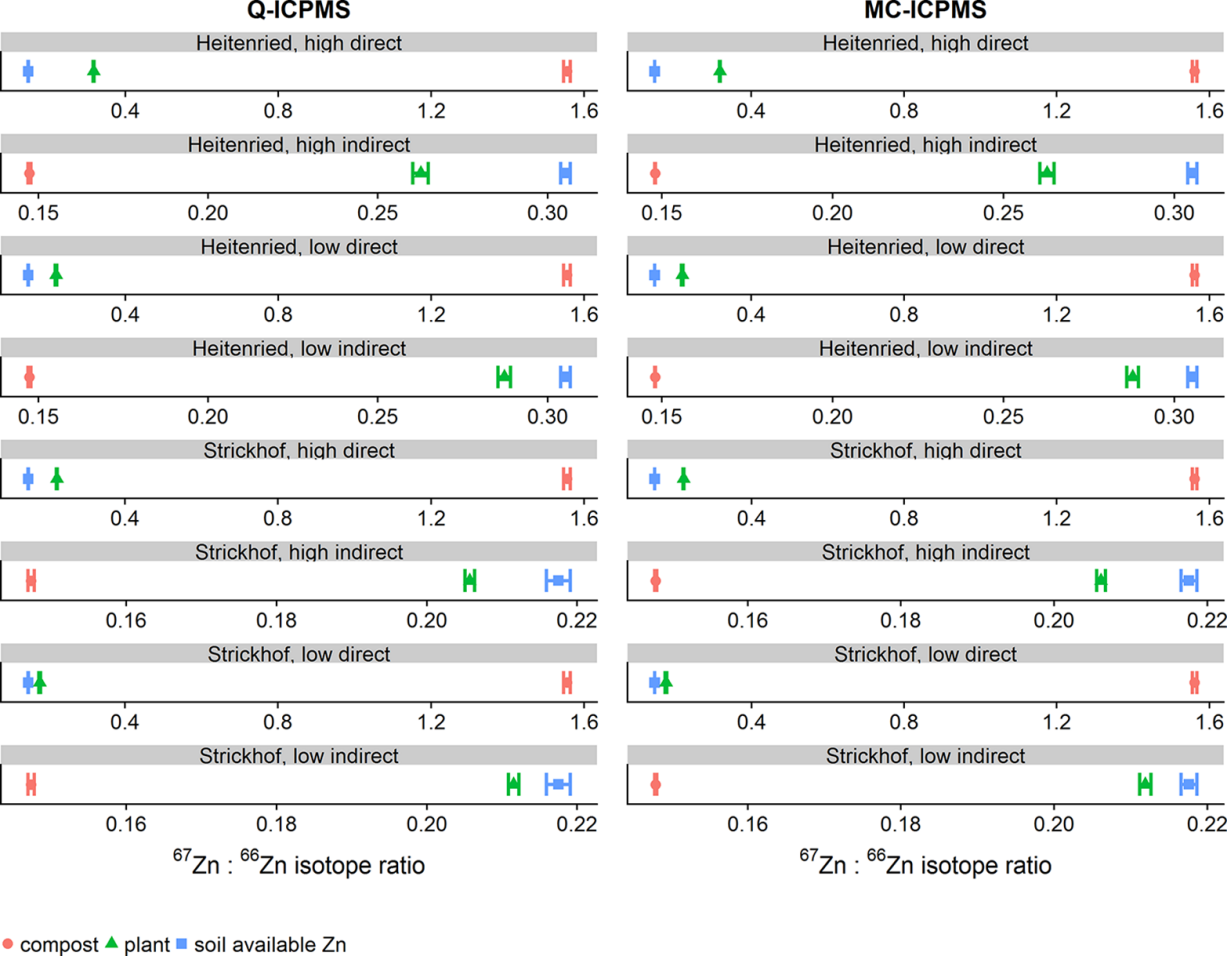
Mean ^67^Zn:^66^Zn-ratios of soil available Zn, the compost, and the plant of purified samples that were measured with Q-ICPMS (left column) or with MC-ICPMS (right column). The ^67^Zn:^66^Zn-ratios of soil available Zn and the plant represent the mean from n = 4 treatment replicates, whereas the ^67^Zn:^66^Zn ratios of the compost represent the mean from n = 4 processing replicates. Error bars represent the 95% confidence interval (CI).

**Figure 1** shows that the ^67^Zn:^66^Zn isotope ratios in the labeled sources were higher in the directly labeled fertilizers (1.561) than in the indirectly labeled soil available Zn pools (0.218 to 0.305). All labeled sources were significantly higher than the measured isotope ratio at natural abundance (0.148). The plant ^67^Zn:^66^Zn ratios were always closer to the ^67^Zn:^66^Zn ratio of the soil than to that of the compost, indicating that soil Zn always provided a larger Zn fraction to the plant than the compost fertilizer. But in all treatments, i.e. direct and indirect labeling, the ^67^Zn:^66^Zn ratio of plant Zn could always be statistically distinguished from the respective ratio of plant available soil Zn, which means that a contribution from the compost was always detectable.

### Comparison of Measurements of Zn Isotope Abundances in the Sources

The analysis of processing replicates from the two sources for ^67^Zn gave no significant differences between Q- and MC-ICPMS measurements (**Table 4**). The confidence intervals were of similar magnitude with both methods for labeled sources, while they were between 3 and 14 times smaller in MC- than in Q-ICPMS analyses of unlabeled sources. With both instruments, the confidence intervals were always larger for labeled than for unlabeled samples. The ^67^Zn abundances of the plants growing on unlabeled soil were close to 4.04% (mole fraction), the natural ^67^Zn abundance reported by the International Union of Pure and Applied Chemistry (Berglund and Wieser, 2011), whereas the abundances of the unlabeled composts were slightly higher (4.12% ^67^Zn).

**Table 4:** ^67^Zn abundances in the sources.

Zinc source	Type	Labeling	Q-ICPMS	MC-ICPMS
			^67^Zn (mole fraction, %)^a^	^67^Zn (mole fraction, %)^a^
Soil	Heitenried	Unlabeled	4.068 ± 0.028a	4.082 ± 0.002a
		^67^Zn-labeled	8.108 ± 0.086a	8.138 ± 0.064a
	Strickhof	Unlabeled	4.048 ± 0.032a	4.072 ± 0.004a
		^67^Zn-labeled	5.909 ± 0.071a	5.937 ± 0.054a
Fertilizer	Wheat straw compost	Unlabeled	4.121 ± 0.022a	4.113 ± 0.007a
		^67^Zn-labeled	31.076 ± 0.203a	31.442 ± 0.169a

a The values for the fertilizer represent the mean and 95% confidence interval of n = 4 processing replicates of ^67^Zn abundances. The values of the soil represent the mean and 95% confidence interval of n = 4 treatment replicates. Letters indicate the significant difference between the two Q-ICPMS and MC-ICPMS measurement technique (pairwise t-test, p-value adjustment method: bonferroni).

### Comparison of ^67^Zn:^66^Zn Isotope Ratios Obtained From Q- and MC-ICPMS Measurements

**Figure 2:**
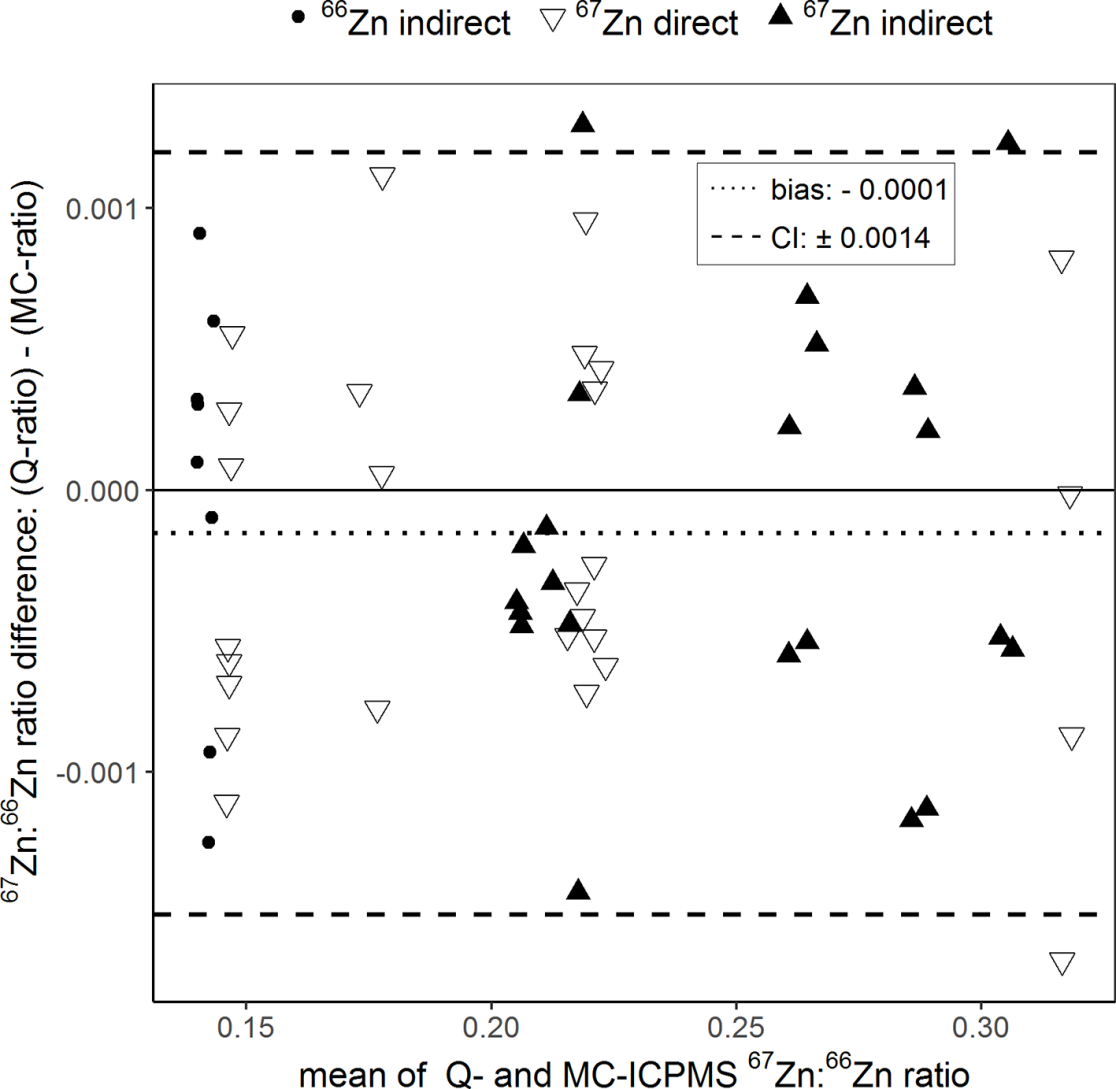
Bland–Altman plot comparing the ^67^Zn:^66^Zn ratios of purified samples that were obtained with single-collector quadrupole ICPMS (Q) and multicollector ICPMS (MC). “Direct” and “indirect” refer to the labeling technique with either an enriched ^66^Zn or ^67^Zn isotope. The results of each sample measured with both instruments were averaged (x-axis) and plotted against their difference (y-axis). The dotted line represents the bias and the dashed lines the 95% confidence interval (CI) of the Bland–Atman analysis.

In total, 57 purified plant samples from the fertilized and reference treatments were analyzed for their Zn isotope ratios with both instruments. Ranging from 0.140 to 0.319, the ^67^Zn:^66^Zn isotope ratios obtained with Q-ICPMS were on average 0.00014 units lower than those obtained with MC-ICPMS (**Figure 2**, all isotope ratios were mass bias corrected). Compared to the range of isotope ratios measured in this study, the average error was equal to ±0.08%. Based on the confidence intervals, the limit of agreement was ±0.0014 units. This means that the isotope ratios obtained with Q-ICPMS can be up to ±0.0014 units higher or lower than the ratios obtained with MC-ICPMS without being significantly different (Giavarina, 2015). Compared to the range of isotope ratios measured in this study, the limit of agreement was equal to 0.68%.

### Comparison of Zndf_fertilizer%_ Values

**Figure 3:**
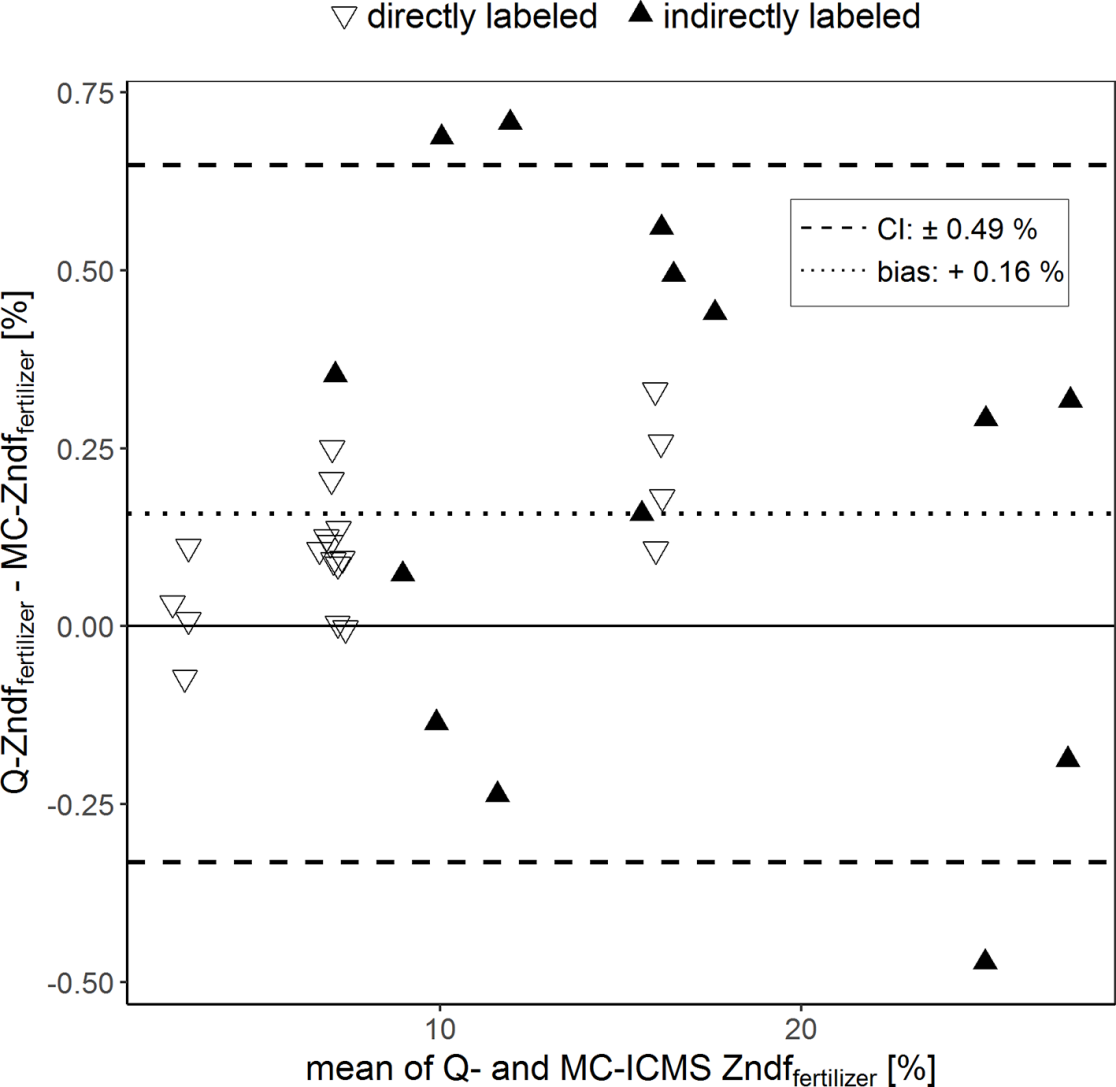
Bland–Altman plot comparing the Zn derived from the fertilizer values (Zndf_fertilizer%_) of purified samples that were obtained with single-collector quadrupole ICPMS (Q) and multicollector ICPMS (MC). The results of each sample measured with both instruments were averaged (x-axis) and plotted against their difference (y-axis). The dotted line represents the bias and the dashed lines the 95% confidence interval (CI) of the Bland–Altman analysis.

The Bland–Altman plot in **Figure 3** compares the percentages of plant Zn derived from the compost (Zndf_fertilizer%_) calculated using the isotope ratios determined with the two ICP-MS instruments. Overall, the Zndf_fertilizer%_ values obtained ranged from 2.6% to 27.6%. In average, the Zndf_fertilizer%_ values obtained with Q-ICPMS were 0.16% higher than those obtained with MC-ICPMS. The limit of agreement indicates that these values could differ up to 0.49%. Furthermore, comparing individual treatments, the Zndf_fertilizer%_ values differed from a minimum of 0.03% to a maximum of 0.50% between the two ICPMS methods.

### Effect of Sample Purification on ^67^Zn:^66^Zn Isotope Ratio Measurements

**Figure 4:**
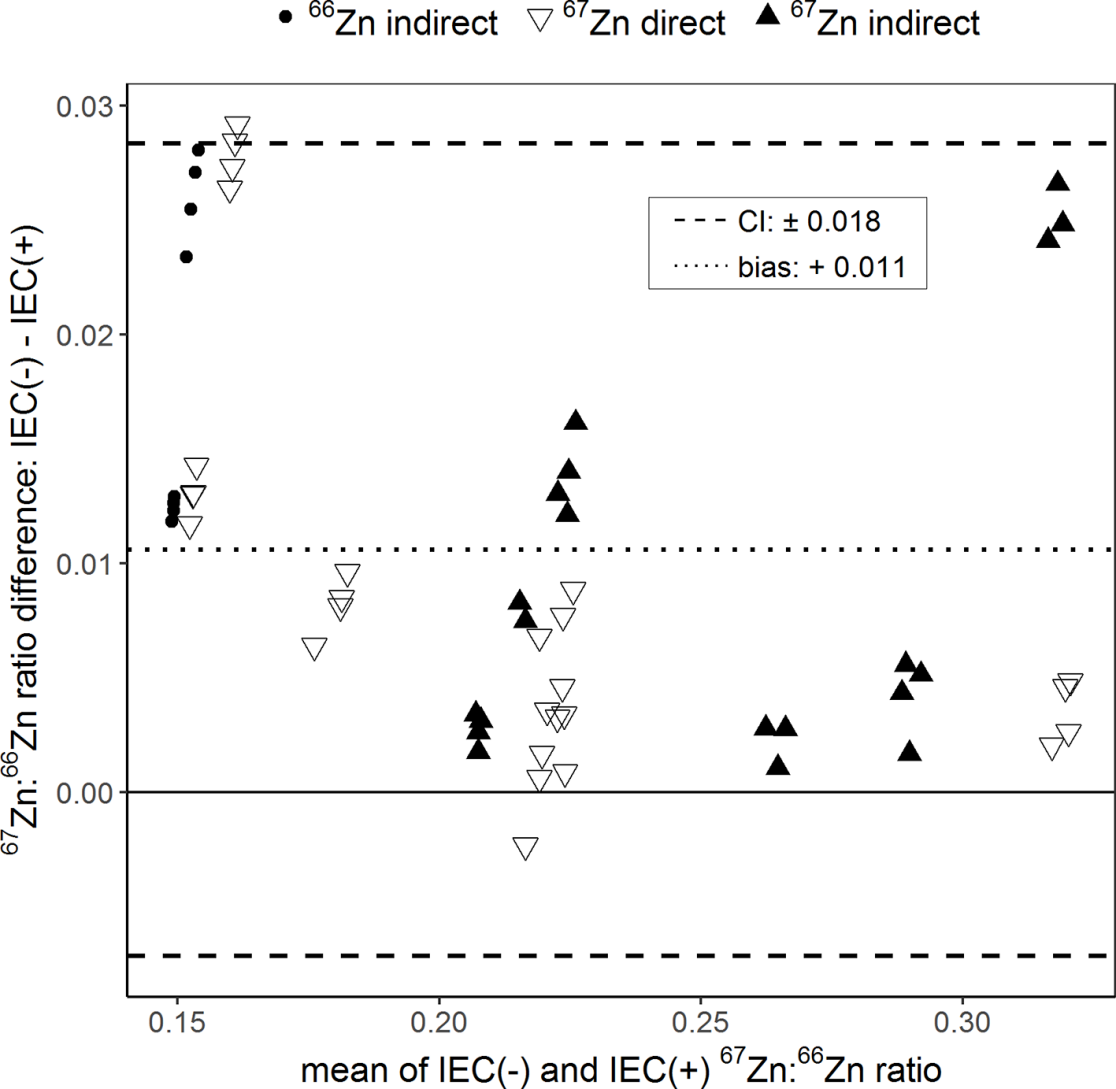
Bland–Altman plot comparing the ^67^Zn:^66^Zn ratios measured in samples for which either ion exchange chromatography (IEC(+)) was conducted to separate the sample matrix from Zn or for which no separation was conducted (IEC(−)). “Direct” and “indirect” refer to the labeling technique with either an enriched ^66^Zn or ^67^Zn isotope. The results of each sample measured with and without IEC were averaged (x-axis) and plotted against their difference (y-axis). The dotted line represents the bias and the dashed lines the 95% confidence interval (CI) of the Bland–Altman analysis.

The Bland–Altman plot in **Figure 4** shows the effect of the sample purification by resin ion exchange chromatography on the determination of ^67^Zn:^66^Zn ratios. Purified and non-purified samples were analyzed only by Q-ICPMS. The ^67^Zn:^66^Zn ratios obtained from the unpurified samples were in average 0.011 units higher than those obtained from the purified samples. This difference was 5.79% of to the total range of isotope ratios measured. Differences in ^67^Zn:^66^Zn ratios between purified and non-purified samples of up to 9.47% (±0.018 units) were still within confidence limits.

## Discussion

### Analytical Precision Versus Experimental Reproducibility

The analytical precision of the ^67^Zn:^66^Zn ratio measurements was in the range of typical RSD for both analytical methods: approximately 1% for Q-ICPMS and 0.01% for MC-ICPMS (Stürup et al., 2008). However, the maximum difference between the final Zndf_fertilizer%_ values obtained with the two methods was 0.49%. A similar relationship between analytical error and variability between sampling and treatment replicates was observed in lead isotope ratios measurements for lichens (Spiro et al., 2004). Hence, the experimental error due to sample preparation and the variability between treatment replicates outweighed the analytical errors associated with the ICPMS analyses. Thus, our study suggests that Q-ICPMS is adequate to trace Zn isotopes in studies on plant Zn sourcing with a limited number of treatment and sample replicates.

### Impact of Sample Matrix on Zn Isotope Measurements

Our results show that Zn separation from the sample matrix by ion exchange chromatography remains an essential step to accurately measure ^67^Zn:^66^Zn ratios with Q-ICPMS (**Figure 4**). Sulfur, magnesium, and nitrogen are abundant major mineral nutrients. Forming argides (magnesium, nitrogen) and oxides (sulfur) in the plasma, they have the potential to affect Zn and Ni (correction of elemental interference) isotope analyses of plant samples by spectral interferences. Furthermore, the sample matrix can affect isotope measurements also through non-spectral effects by influencing sample transport efficiency, ionization, ion transfer, and ion detection (Prohaska et al., 2014).

Whether the time-consuming purification step is needed might depend on the degree of isotope enrichment, the concentration of the target element, and on the analytical device used for the isotope analysis. With indirect source tracing, it was not feasible to enrich the isotope as much as with direct source tracing (**Figure 1**). Thus, sample purification might be essential with indirect source tracing (see also *Comparison of* *^67^* *Zn:* *^66^* *Zn Isotope Ratios Obtained From Q- and MC-ICPMS Measurements*). If Zn isotope source tracing method is used e.g. in Zn biofortification studies, Zn isotopes may have to be analyzed in samples with low Zn concentrations, and thus relatively large spectral interferences and matrix effects may have to be considered. An additional benefit of sample purification is that it allows at the same time to study other elements besides Zn with lower concentrations in soil–plant systems. For instance, the non-essential and highly toxic element Cd, which behaves similar as Zn in soil–plant systems, is usually about 100 to 300 times less concentrated than Zn in non-contaminated soils (Mertens and Smolders, 2013). Previous studies successfully measured Zn isotope ratios of unpurified samples by using a high-resolution single-collector ICPMS (HR-ICPMS) to determine isotope exchange kinetics for Zn in soils (Sivry et al., 2011; Ren et al., 2016). These double-focusing magnetic sector instruments have a higher resolving power than Q-ICPMS and are better in resolving spectral interferences (Thomas, 2013). However, it needs to be tested if HR-ICPMS can resolve spectral interferences of samples with low Zn concentrations and low isotope enrichment as in the case of indirect source tracing in our study.

### Indirect Source Tracing

Indirect source tracing enables to test the efficiency of complex organic fertilizers for Zn biofortification. Critical issues for this method are on the one hand that the amount of added enriched stable isotope needs to be large enough to make the sources distinguishable, while on the other hand the mass of Zn added to the soil must not be so large that the plant available soil Zn is altered significantly. To assess if the isotope enrichment in the labeled source was large enough to resolve the Zn source contributions to the plant, we compared the isotope enrichment with the experimental precision (**Table 5**). The ^67/66^Zn isotope enrichment in the directly labeled systems was equal to ^67/66^Zn_labeled.source_ − ^67/66^Zn_unlabeled.source_ = 1.4089. If we divide this value by using a conservative estimate of 2 standard deviations (2sd) of the experimental precision that was obtained by measuring the ^67/66^Zn isotope ratio in the plants, the result is that 414 ^67/66^Zn isotope ratios could be statistically distinguished from each other in between the two Zn sources. This resolution means that Zn contributions of approximately 0.24% (100% divided by 414) from the sources to the plant can be detected. In the indirectly labeled sources, the isotope enrichment was 0.0702 in the Strickhof and 1.579 in the Heitenried soil. With this isotope enrichment, only n = 26 and 58 ^67/66^Zn isotope ratios can be statistically distinguished from each other in the Strickhof and Heitenried soil, respectively. With this resolution, Zn contributions of approximately 1.72% to 3.85% from the sources to the plant can be detected. The weaker resolution in the indirectly labeled sources was caused by dilution of the ^67^Zn enriched isotope label in the pool of resident soil Zn. Furthermore, the isotope enrichment of the soil available Zn pools differed between the soils, and thus also the number of significantly distinguishable isotope ratios. Previous studies showed that soils can contain different number of compartments which have distinct Zn isotope exchange rates and contain distinct quantities of Zn (Cobelli et al., 2000; Diesing et al. 2008). Hence, these compartments determine the mass of Zn in the soil available Zn pool and thus also the mass of ^67^Zn that needs to be added to a soil to sufficiently enrich the soil available Zn pool with ^67^Zn.

**Table 5:** Resolution of Zn source contribution.

Labeling approach	Labeled Zn source	Zn sources	^67^Zn:^66^Zn isotope ratio^a^	Isotope enrichment^b^	Experimental precision^c^	Significantly distinguishable isotope ratios^d^
			mole mole^−1^	mole mole^−1^	mole mole^−1^	−
Indirect	Heitenried soil (acidic)	Compost	0.1473	0.1579	0.0027	58
		Soil available Zn	0.3052			
	Strickhof soil (alkaline)	Compost	0.1473	0.0702	0.0027	26
		Soil available Zn	0.2175			
Direct	Wheat straw compost	Compost	1.5552	1.4089	0.0034	414
		Soil available Zn	0.1463			

a Mean isotope ratios of n = 4 treatment (soil available Zn) or processing replicates (compost). Isotope ratios were determined with Q-ICPMS.

b Calculated as: isotope enrichment = ^67/66^Zn_labeled.source_− ^67/66^Zn_unlabeled.source_.

c Average of 95% confidence interval of the experimental precision (2sd of n = 4 treatment replicates) of either sources or sinks.

d Calculated as: n = isotope enrichment : experimental precision.

**Figure 5:**
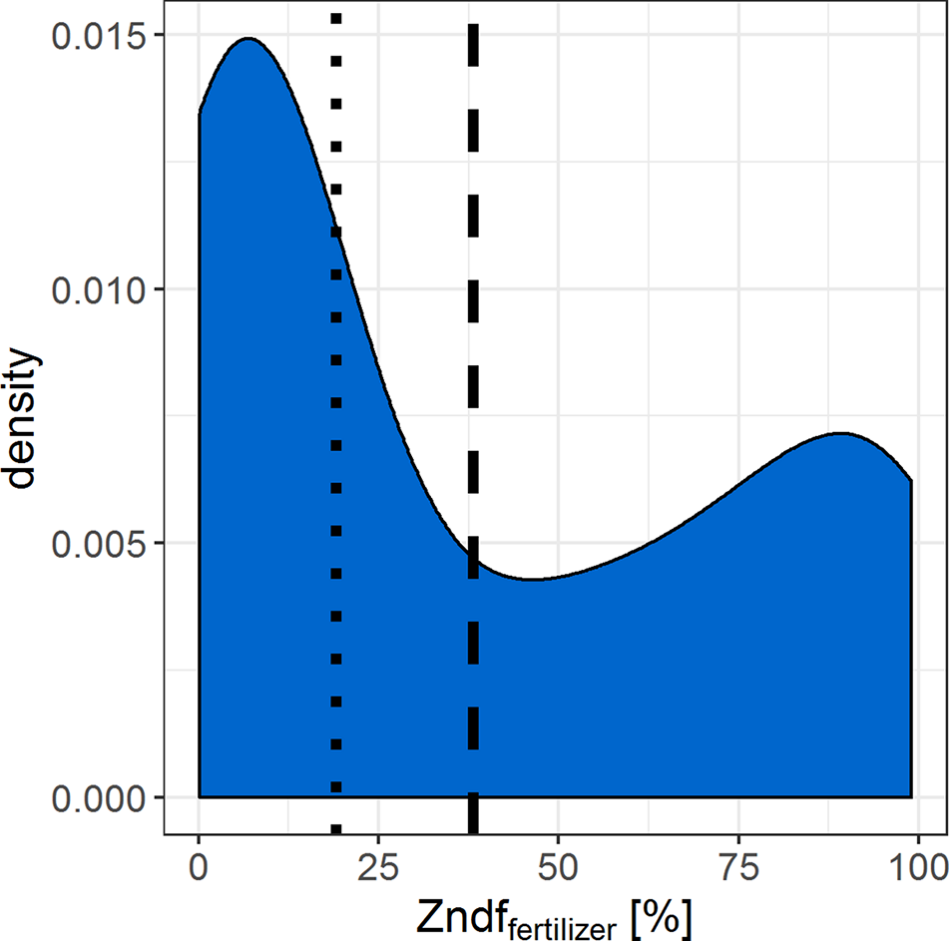
Density curve that summarizes the Zn derived from fertilizer values (Zndf_fertilizer%_) found in similar Zn source tracing studies (Amer et al. 1980; Nanzer, 2012; McBeath et al., 2013; Aghili et al., 2014; McBeath & McLaughlin, 2014). Dashed line = median, dotted line = mean of n = 101 Zndf_fertilizer%_ values.

If the resolution of n = 26–58 statistically distinguishable isotope ratios between the two Zn sources is sufficient to determine the Zn contributions of the sources depends on the range of expected Zndf_fertilizer%_ values. We compiled data from similar Zn source tracing studies that cover several soil and fertilizer types (**Figure 5**). The Zndf_fertilizer%_ values ranged from 0.1% to 99% with an average of 38% and a median of 19%. The Zndf_fertilizer%_ values >50% were mostly reported for Zn sources that readily dissolved in soils such as Zn sulfates and Zn oxides (Nanzer, 2012; McBeath and McLaughlin, 2014). Studies that tested organic fertilizers or complex recycling fertilizer reported values below 50% (Amer et al., 1980; Nanzer, 2012; Aghili et al., 2014). Thus we expect that the majority of Zndf_fertilizer%_ values, and their corresponding ^67^Zn:^66^Zn isotope ratios, range upto 50%. Within this range, 13 and 29 statistically distinguishable isotope ratios can be resolved with the obtained experimental precision of ^67^Zn:^66^Zn = 0.0027 (2sd). To improve this resolution, more ^67^Zn or a less abundant Zn isotope such as ^70^Zn needs to be added to the soil to increase the isotope enrichment without further increasing the mass of Zn added to the soil (Larner and Rehkämper, 2012).

For future experiments, we recommend that the optimal mass of enriched ^67^Zn or ^70^Zn label that is added to a soil is determined in pretests, in which e.g. Zn isotope composition of available soil Zn is determined after adding different amounts of enriched Zn isotopes to the soil. Furthermore, available soil Zn pools should be assessed before and after labeling by using e.g. salt extracts, chelating agents, DGT techniques, or isotope exchange techniques (Zhang et al., 2004; Menzies et al., 2007; Diesing et al., 2008; Ren et al., 2016) to test the impact of soil labeling on the size of the soil available Zn pool.

## Conclusions

We tested the use of Q-ICPMS in comparison to the more precise but less accessible MC-ICPMS as analytical method to determine plant Zn uptake from complex organic fertilizers using stable Zn isotope source tracing. We found that the analytical precision was by far sufficient in relation to sampling and experimental variability and that also the bias relative to the MC-ICPMS measurements was negligible for the calculation of plant Zn fractions derived from the fertilizer source. For indirect source tracing, it is recommended to purify samples by ion exchange chromatography prior to Q-ICPMS analysis. Prior to the indirect source tracing experiment, stable isotope labeling of soil available Zn needs to be optimized for each soil by maximizing the isotope enrichment of this Zn source while not changing the plant availability of Zn in the soil.

## Data Availability Statement

All datasets generated for this study are included in the article/supplementary material.

## Author Contributions

TD-A conducted the laboratory work. WM and DW were leading the write-up of the manuscript while all co-authors provided inputs. EF initiated the project, CZ helped to conduct the MC-ICPMS measurements, DW advised the comparison of the two instruments, and EF and RS supervised the project.

## Funding

The project was funded by the Swiss State Secretariat for Education and Research within the framework of the COST (European Cooperation for Science and Technology) action “Mineral-Improved Crop Production for Healthy Food and Feed” (FA0905) with the project number C10.0085.

## Conflict of Interest

The authors declare that the research was conducted in the absence of any commercial or financial relationships that could be construed as a potential conflict of interest.
